# Supplementary material for: Semi-orthogonal subspaces for value mediate a tradeoff between binding and generalization
Source: ArXiv. 2023 Sep 14:arXiv:2309.07766v1. Preprint. [Version 1] (PMC10516109)
Supplement: 1 [file NIHPP2309.07766V1-supplement-1.pdf]

Supplementary Figures

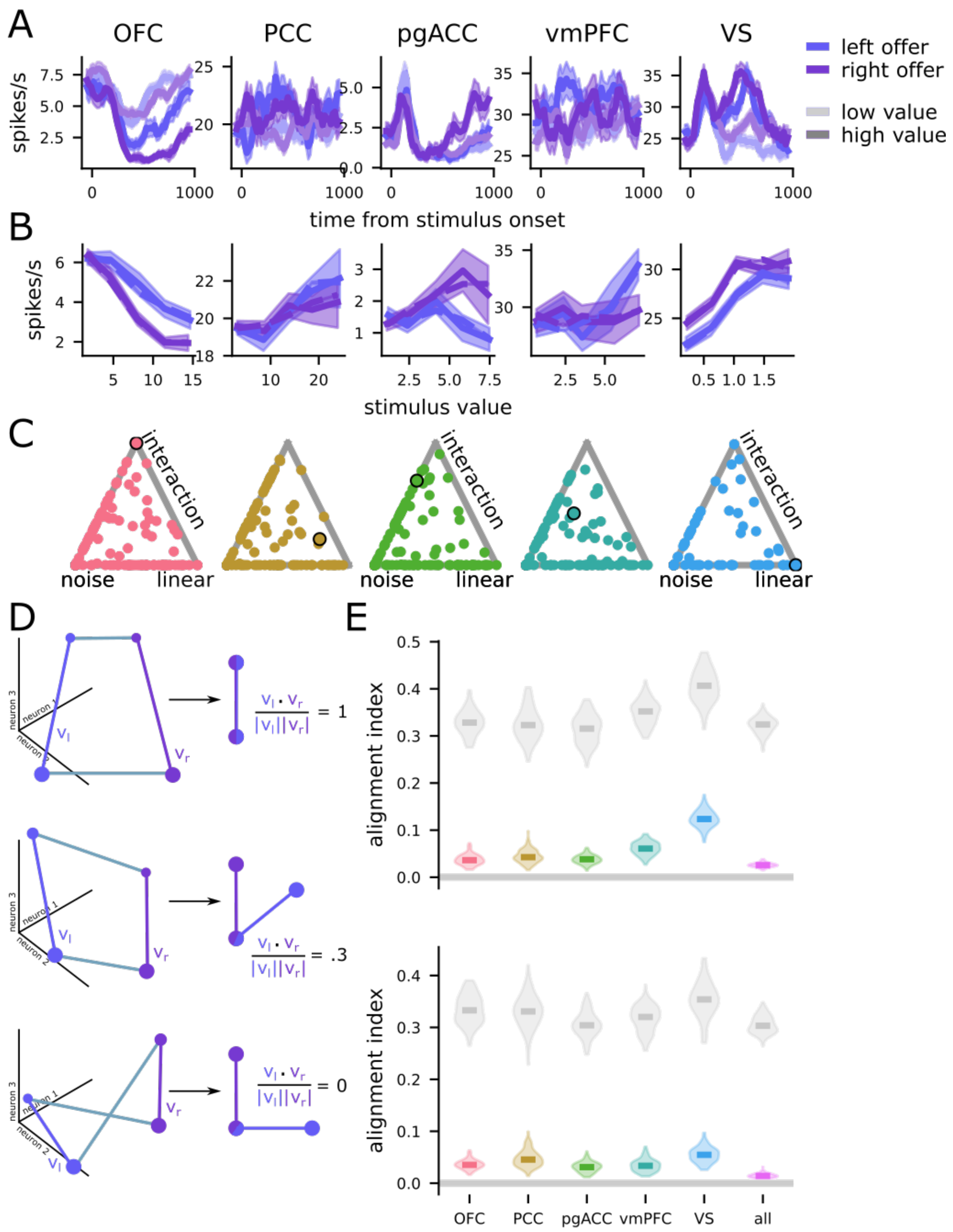

Figure S1. Example neurons, model comparison, and subspace correlations for the linear models with a nonlinear value representation. **A.** The firing rates of example neurons from each region during the offer window, shown for high and low value offers presented on the left or right side (100 ms boxcar filter, shaded area is SEM). **B.** The value-response function for each neuron in **A**. The value-response function fit by the linear regression model with a b-spline value encoding and an interaction term is overlaid (dashed lines). The b-spline representation uses 4 knots and is degree 2. **C.** A simplex showing the weight given to each of the noise-only, linear, and interaction regression models by the Bayesian model stacking analysis. The points corresponding

to the example neurons shown in **A** and **B** have dark outlines here. Both the linear and interaction categories include both linear and spline value representation models. **D.** Schematic of three different representational geometries that would lead to different subspace correlation results. (top) Two perfectly aligned value vectors v_l and v_r in population space (left) would produce a subspace correlation close to 1 (right). (middle) Two partially aligned value vectors v_l and v_r in would produce a subspace correlation between 0 and 1 (note there is an additional possibility: partially aligned but negatively correlated subspaces; not schematized). (bottom) Two unaligned value vectors v_l and v_r would produce a subspace correlation close to 0. **E.** Alignment indices for all regions for the offer presentation window. The gray point is the subspace correlation expected if the left- and right value subspaces were aligned and corrupted only due to noise. **E.** Same as **D.** for the delay period.

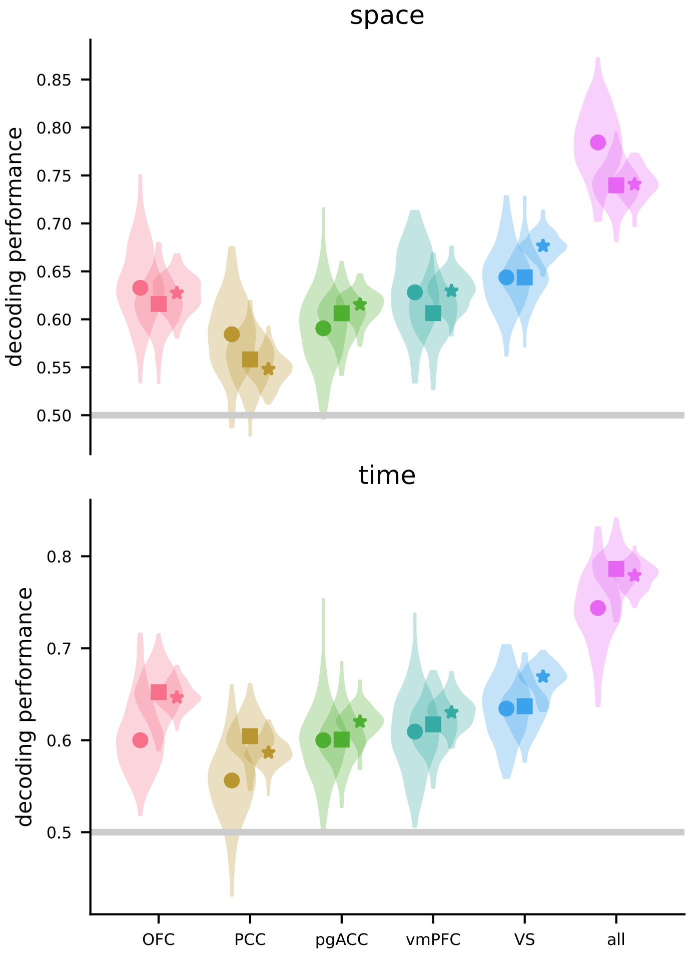

Figure S2. Value decoding, value generalization, and predicted value generalization of the code within each recorded region. **A.** Pseudopopulation value decoding performance (circles), generalization performance (squares, trained on offers from one side, tested on offers from the other side), and predicted generalization performance (stars) shown for each region and the neural population combined across regions ("all"), shown for the left and right value comparison. The violin plot shows the values produced from two hundred bootstrap resamples of the trials. **B.** The same as **A** except shown for the offer 1 and offer 2 comparison. PCC did not have the required number of trials (160) for each condition.

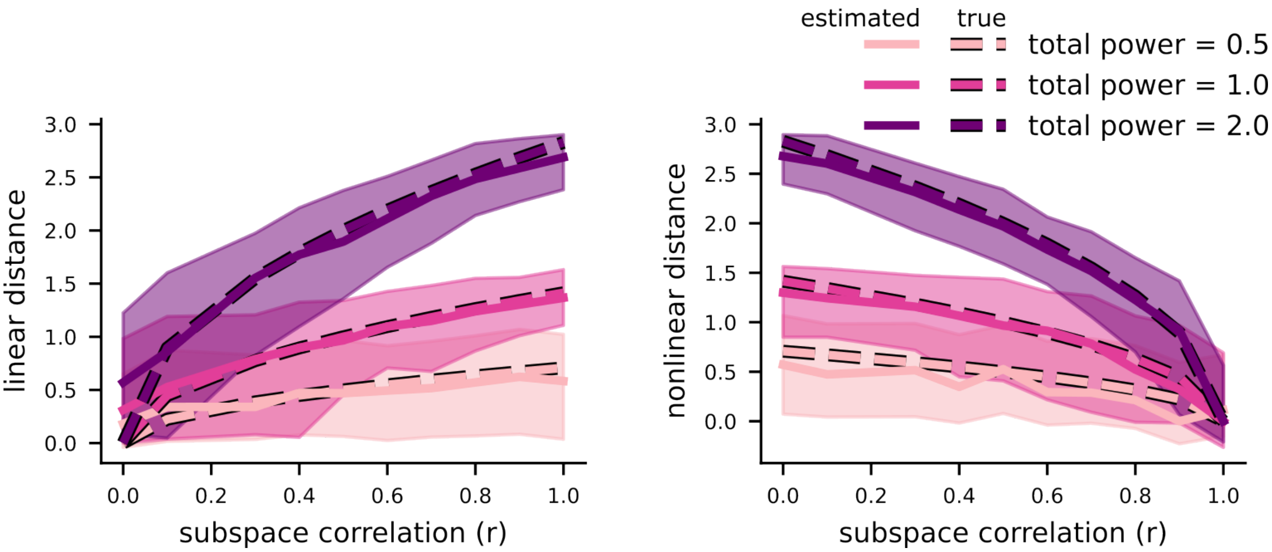

Figure S3. Accurate recovery of linear and nonlinear distances from simulated data. The true (dashed line) and estimated (solid line with error bars) linear (left) and nonlinear (right) distances from simulated data. Our decomposition accurately recovers the linear and nonlinear distances, as the true value is always within error bars of the estimated value and the estimate is typically unbiased.

Neurons with heterogeneous representations of value across positions drive subspace separation

What is the single neuron basis of the subspaces? One simple possibility is that the subspaces may be composed almost entirely of nonlinearly selective neurons that each only represent the value of offers at a single spatial position (i.e., neurons with canonical spatial receptive fields). The full population, then, would be composed of two largely separate subpopulations: one for offers on the left and another for offers on the right (**Figure S4A**, left, and S**4B**, top). This organization could also be viewed as a gain modulation code, where spatial position strongly modulates the value code of single neurons, without changing their tuning (e.g., **Figure S4E**, left: a left offer tuned cell). Alternatively, subspace separation could be achieved by neurons with heterogeneous nonlinear responses to offer value and position, and that contribute activity to multiple subspaces (e.g., **Figure S4E**, right; Fusi et al., 2016; Tang et al., 2020). We refer to this as the *shared population hypothesis* (**Figure S4A**, right, and S**4B, bottom**).

These different coding strategies lead to distinct predictions across the population. If the population was dominated by cells that respond more strongly to offers presented at one of the two positions (or even cells that respond only to offers presented at one position), then we would expect a bimodal distribution of differences in selectivity for left and right value across the population (**Figure S4C;** Elsayed et al., 2016). Alternatively, if the population is composed of neurons that represent value differently across the two positions, but with similar strength, then we would expect this distribution to be unimodal (**Figure S4C**). We discriminated between these two possibilities using the regression coefficients to characterize each neuron's value representation for each location. We then asked if the distributions of firing rate differences diverged from a unimodal distribution using Hartigan's dip test. We then repeated this analysis

for all time-windows. We found no evidence for bimodality (*p* > 0.9 for all areas and subjects). The first offer on time window provides an example of the typical distribution (**Figure S4D)**. This result therefore supports the idea that the value of left and right offers are encoded in distinct subspaces, but not in distinct populations of neurons (as would be expected by a simple spatial receptive field model). Note that this finding of non-categoricality is consistent with several other recent studies emphasizing non-categorical neural responses (Blanchard et al., 2018; Raposo et al., 2014; Kaufman et al., 2022).

The lack of bimodality implies there may be several types of nonlinear encoding neurons that drive the subspaces. Next, we show the left and right subspaces are supported by neurons with both spatially-tuned gain modulation and heterogenous value representations (e.g., **Figure S4E)**. Specifically, we quantified the proportion of each neuron type in each brain region. First, we searched for neurons that contributed to both a left and right subspace within an analysis epoch, using a measure of subspace contribution (Xie et al., 2022). The subspace contribution effectively computes the proportion of variance a neuron contributes to a subspace. Next, remaining neurons were classified based on whether their preferred value was either constant (gain modulated) or shifted across subspaces (heterogeneous nonlinear). Examining the response profiles of neurons meeting the multi-subspace criterion shows both those with gain modulation and those with shifting tuning to value that depends on the subspace (**Figure 4E**). Averaging across all regions and windows, we found that both types were found in roughly equal proportions. These results rule out simple spatial subpopulation or wide-spread gain modulation explanations of value subspaces and support the idea that the two subspaces are represented in the same population of neurons, but semi-orthogonal axes in population space.

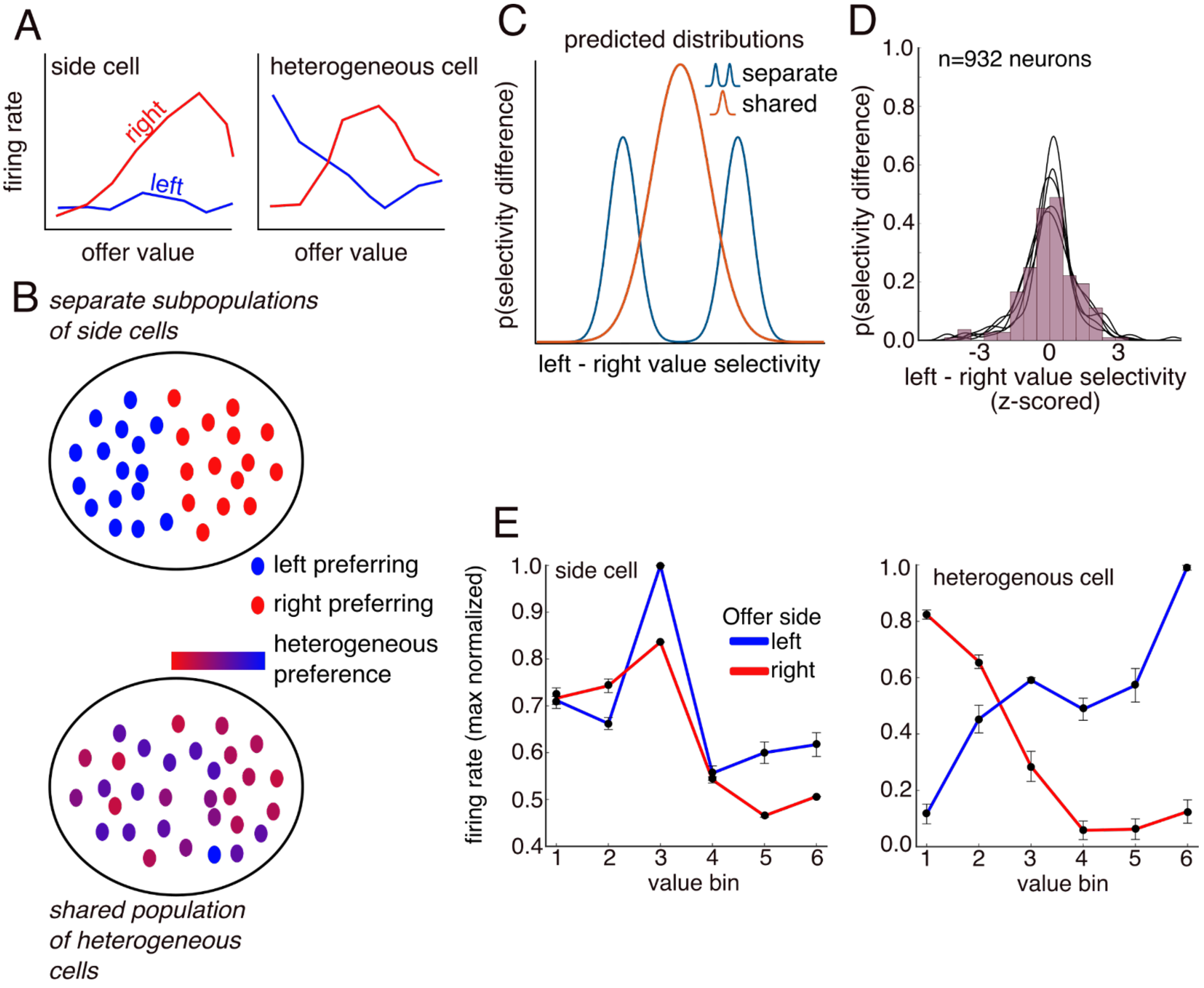

Figure 4. Understanding the nonlinear selectivity underlying subspaces and binding. **A.** Two schematic kinds of nonlinear selectivity: (left) A strong side preference and (right) two different response profiles to the two different sides. **B.** The two kinds of selectivity give rise to two distinct hypotheses for selectivity across the population: (top) Separate subpopulations, each composed of cells with a strong preference for one of the two positions and (bottom) a single population composed of cells with heterogeneous response profiles for the two sides. Both forms of nonlinear population selectivity achieve subspace binding. (**C**). These hypotheses (**A-B**) predict differences in how the distribution of selectivity differences for left and right value subspaces will appear. The separate subpopulations hypothesis predicts a closer to bimodal distribution (blue line), while the shared, heterogeneous hypothesis predicts a unimodal distribution (orange line). (**D**) estimated distribution of differences in value selectivity for left and right subspaces for offer 1 on time window. Each line is a different region, showing they are all unimodal. (**E**). Example OFC nonlinear encoding neurons showing: one has a weak side preference (left) and the other has a heterogeneous response profile (right).
